# Application of the adaptive Monte Carlo method for uncertainty evaluation in the determination of total testosterone in human serum by triple isotope dilution mass spectrometry

**DOI:** 10.1007/s00216-024-05380-z

**Published:** 2024-06-19

**Authors:** Gongcheng Liu, Huimin Wang, Yanlin Han, Chunlong Liu, Man Liang

**Affiliations:** 1 Reference Laboratory, Autobio Diagnostics Co., Ltd, 199 Fifteenth Street, National Eco &Tech Development Zone, Zhengzhou, 450016 Henan China; 2 Department of Laboratory Medicine, Affiliated Hospital of Nantong University, Nantong, 226001 Jiangsu China

**Keywords:** The adaptive Monte Carlo Method, Uncertainty evaluation, Testosterone, Triple isotope dilution, Mass spectrometry

## Abstract

The measurement uncertainty is a crucial quantitative parameter for assessing the reliability of the result. The study aimed to propose a new budget for uncertainty evaluation of a reference measurement procedure for the determination of total testosterone in human serum. The adaptive Monte Carlo method (aMCM) was used for the propagation of probability distributions assigned to various input quantities to determine the uncertainty of the testosterone concentration. The basic principles of the propagation and the statistical analysis were described based on the experimental results of the quality control serum sample. The analysis of the number of Monte Carlo trials was discussed. The procedure of validation of the GUM uncertainty framework using the aMCM was also provided. The number of Monte Carlo trials was 2.974 × 10^6^ when the results had stabilized. The total testosterone concentration was 16.02 nmol/L, and the standard uncertainty was 0.30 nmol/L. The coverage interval at coverage probability of 95% was 15.45 to 16.62 nmol/L, while the probability distribution for testosterone concentration was approximately described by a Gaussian distribution. The validation of results was not passed as the expanded uncertainty result obtained by the aMCM was slightly lower, about 7%, than that by the GUM uncertainty framework with consistent results of the concentration.

**Graphical Abstract:**

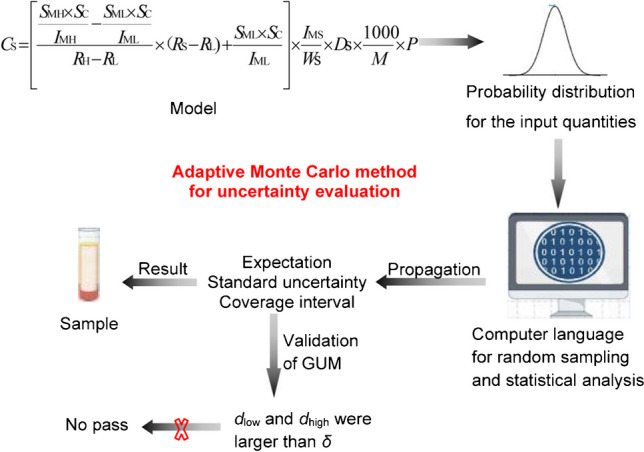

**Supplementary Information:**

The online version contains supplementary material available at 10.1007/s00216-024-05380-z.

## Introduction

No measurement is absolutely accurate due to the influence factors such as measurement procedure, calibrated measuring system and measurement conditions. The true quantity values are unknowable in principle and in practice due to the impact of random and systematic effects on the analysis [1]. An coverage interval that may be expected to encompass a large fraction of the distribution of values that could reasonably be attributed to the quantity subject to measurement is necessary to provide for the measurement result [2]. The measurement result is only complete expressed as an estimate of the true value associated a non-negative parameter characterizing the dispersion of the quantity value being attributed to a measurand, called measurement uncertainty. As a quantitative indication of the quality of the result, the measurement uncertainty is crucial for the reliability of the measurement results, for the evaluation of analytical procedures, for comparing measurement results with reference values given in a specification and for making appropriately decision. Relevant standards and documents clearly require the measurement uncertainty to be given for reference measurement results and reference materials [2–4].

The evaluation of measurement uncertainty is a process that propagate the probability distribution of input quantity through the measurement model to obtain the probability distribution of output quantity by a specific method. The examples of uncertainty evaluation is mainly based on *Guide to the Expression of Uncertainty in Measurement* (GUM) since it was published by International Organization for Standardization (ISO) in 1993 [2]. As an approximate analytical method, GUM is based on the law of propagation of uncertainty assigned to various input quantities. To ensure the estimate of the output quantity and the associated measurement uncertainty are reliable and the coverage interval is realistic, GUM need the measurement model to be linear or linearizable and the characterization of the measurand to be a Gaussian distribution or a scaled and shifted *t*-distribution.

A detailed guidance was proposed by the Joint Committee for Guides in Metrology for the aspects of uncertainty evaluation that is not explicitly treated in the GUM, known as Monte Carlo Method (MCM) [5]. As a practical alternative to the GUM, the MCM is extremely useful in situations where the conditions for the GUM are not fulfilled, or it is unclear whether they are fulfilled. *Quantifying uncertainty in analytical measurement* (QUAM) introduce MCM in its E3 and consider MCM as a validation method to check whether the GUM is applicable because of the complexity of the model [6]. The MCM is recommended for the evaluation of measurement uncertainty of reference materials according to ISO Guide 35 [7].

The MCM is a general numerical simulation approach that the propagation of probability distributions assigned to various input quantities is implemented through a mathematical model of measurement and a statistical method depend on the random and repeated sampling for the evaluation of measurement uncertainty. The MCM has been widely used in the field of error analysis, drug metabolism, prediction model, and evaluation of reference interval [8–11]. The MCM has also be a research hotspot in uncertainty evaluation in the fields of physical and chemical metrology in recent 20 years, especially with the application of computer and commercial software [12–18]. There are only a few reports of the implementation in clinical medical measurement [19, 20].

Isotope dilution mass spectrometry (IDMS) is widely recognized as one of the most accurate methods for high-order reference measurement procedure (RMP) and has been witnessed many elaborations and developments over the years [21–25]. However, only a few publications dealt with IDMS measurement uncertainty [26–30]. In most of these publications, the measurement uncertainties were determined according to the relative standard uncertainties of several main factors. We reported the uncertainty evaluation of the RMP for total thyroxine in human serum in strict accordance with GUM [31].

Testosterone is the key male sex hormone which regulates sexual differentiation and promotes first and secondary sexual characteristics [30]. The high-order RMPs for total testosterone in human serum are based on IDMS according to the Joint Committee for Traceability in Laboratory Medicine (JCTLM) database. As a criteria for the reliability of the RMPs for total testosterone in human serum, the measurement uncertainties were calculated by combining the relative standard uncertainties of several main factors [30, 32, 33]. In this study, the aMCM was proposed for the uncertainty evaluation of a RMP for total testosterone in human serum.

## Materials and methods

A RMP for the determination of total testosterone in human serum, based on triple isotope dilution liquid chromatography tandem mass spectrometry, has been performed and critically evaluated in our laboratory. The nomination of the RMP for reference measurement services had been reviewed by the JCTLM and was recently approved for listing in the JCTLM database. The report for the details of isotope dilution mass spectrometry procedure and the data of method validation was provided in the JCTLM database. The lyophilized serum material, Hormone Marker Control II obtained from Bioyuan Biotechnology Co., Ltd., was used as a quality control serum sample after reconstitution.

### Principles of uncertainty evaluation with aMCM

The MCM is regarded as a numerical simulation method. The key of the method is repeated sampling from the probability density function (PDF) of each input quantity and calculating the output quantity according to the mathematical model in each case. Then, the statistical analysis of the calculation results is carried out for the determination of the probability distribution for the output quantity. The properties of output quantity such as expectation, variance, and coverage intervals are obtained.

#### Formulation

The evaluation of measurement uncertainty is based on the description of the relationship between the measurand and the different variables that affect the value of the measurand, called input quantities [2]. A measurand *Y* is determined from *N* relating quantities *X*_1_, *X*_2_, …, *X*_*N*_ through a functional relationship *f* as Eq. (1).

1 $$Y=f\text{(}{X}_{1}\text{, }{X}_{2}\text{, }...\text{ , }{X}_{N}\text{)}$$

The mathematical model is related to the principles of measurement, incorporate corrections, and other effects that influence the measurement.

The PDF of each input quantity is assigned based on experimental data, literature, metrological knowledge, expert experience, historical data, and other relevant information according to Bayes’ theorem or the principle of maximum entropy. The PDF expresses the state of knowledge about the quantity which should include at least the expectation, standard uncertainty, and probability distribution type. A rectangular distribution R(*a*, *b*) over the interval [*a*, *b*] would be assigned to a quantity *X* according to the lower limit *a* and the upper limit *b* with *a* < *b*. A Gaussian probability distribution N(*x*, *u*^2^(*x*)) would be assigned to a quantity *X* according to the best estimate *x* and associated standard uncertainty *u*(*x*).

#### Propagation

In the implementation of MCM, the statistical random sampling from the PDFs of the input quantities *X*_*N*_ is carried out. The number *M* of Monte Carlo trials is selected for the model evaluation. With *x*^(*j*)^_1_,* x*^(*j*)^_2_,…,*x*^(*j*)^_*N*_, *j* = 1,…,*M*, the model value *Y* is obtained according to Eq. (2).

2 $${y}^{(j)}=f\text{(}{{x}_{1}}^{(j)}\text{, }{{x}_{2}}^{(j)}\text{, }...\text{ ,}{ \, {x}_{N}}^{(j)}\text{)}$$

The statistical analysis is conducted on the *M* model values of *Y*, *y*^(1)^, *y*^(2)^, …, *y*^(*M*)^, to determine the probability distribution for the output quantity. The expectation of *Y* is estimated by the average of the *M* model values as Eq. (3).

3 $$\overline{y }=\frac{1}{M}\sum_{j=1}^{M}{y}^{(j)}$$

The standard uncertainty *u*(*y*) is estimated by the standard deviation of the *M* model values as Eq. (4).

4 $$u(y)=\sqrt{\frac{1}{M-1}\sum_{j=1}^{M}{({y}^{(j)}-\overline{y })}^{2}}$$

The model values, *y*^(1)^, *y*^(2)^, …, *y*^(*M*)^, are sorted into strictly non-decreasing order. The 95% coverage interval [*y*_low_, *y*_high_] for *Y* can be obtained by taking the 2.5 and 97.5 percentiles of the sample order.

The number of Monte Carlo trials has a big impact on “shape” of the PDF for the output quantity and on the coverage probability. The closeness of agreement between the numerical results and the expectation is expected to be proportional to *M*^−1/2^ [5]. The estimate of output quantity and the associated standard uncertainty are closer to the true quantity values as the number of *M* increases, but the calculation takes longer. It is chosen a priori. The number of *M* is at least 10^4^ times greater than 1/(1-*p*) in general. However, there is no guarantee that any specific pre-assigned number will suffice. A procedure for selecting the number of Monte Carlo trials adaptively, known as aMCM procedure, is implemented to obtain the appropriate number. A property of aMCM is that the *M* taken is economically consistent with the expectation of achieving a required numerical tolerance *δ* related to the number of significant decimal digits regarded as meaningful in a numerical value [5].

The implementation of aMCM involves carrying out an increasing the number of Monte Carlo trials until the various results have stabilized in a statistical sense. *y*(*h*), *u*(*y*(*h*)), *y*(*h*)_low_, and *y*(*h*)_high_ as an estimate of *Y*, the associated standard uncertainty and the left- and right-hand endpoints of a 95% coverage interval are calculated according to Eqs. (3) and (4) for the *h*th application of MCM with carrying out *M* Monte Carlo trials in the sequence. The standard deviation *s*_*y*_ associated with the average of the estimates *y*(1),..., *y*(*h*) of *Y* is estimated as Eq. (5).

5 $$s_{y}=\sqrt{\frac{1}{h(h-1)}\sum_{r=1}^{h}(y(r)-y{)}^{2}}$$

where

$$y=\frac{1}{h}\sum_{r=1}^{h}y(r)$$

The counterpart standard deviations *s*_*u*__(__*y*__)_, *s*_*y*__l__o__w_, and *s*_*y*__h__i__g__h_ associated with the standard uncertainty and the left- and right-hand endpoints of 95% coverage interval are also calculated. The results of *s*_*y*_, *s*_*u*__(__*y*__)_, *s*_*y*__l__o__w_, and *s*_*y*__high_ are deemed to have stabilized if twice the standard deviations are less than *δ*. If any of 2*s*_*y*_, 2*s*_*u*__(__*y*__)_, 2*s*_*y*__low_, and 2*s*_*y*__high_ exceeds *δ* or *h* is 1, the number of application of MCM in the sequence increases by one until the various results have stabilized. Then, all *h*×*M* model values obtained are used to the calculate estimate *y*,* u*(*y*) and 95% coverage interval.

The propagation involved complex calculation process is operated with computer software or computer language such as MATLAB and Python now.

### Validation of the GUM uncertainty framework using the aMCM

A coverage interval *y* ± *Up* at coverage probability of* p* for the output quantity is yield according to the GUM. The endpoints of the coverage interval at coverage probability of* p*, *y*_low_, and *y*_high_ are provided according to the aMCM procedure. The absolute differences of the respective endpoints of the required coverage interval by GUM and aMCM are calculated as Eqs. (6) and (7).

6 $${d}_{\text{low}}=\left|y-Up-{y}_{\text{low}}\right|$$

7 $${d}_{\text{high}}=\left|y+Up-{y}_{\text{high}}\right|$$

If both the absolute differences *d*_low_ and *d*_high_ are no larger than *δ*, the comparison is favorable, and the GUM has been validated.

## Result

### Measurement model

Similar to our previous work [31], the measurement model is shown as Eq.(8), with several different parameters defined as Table 1.

8 $$C{_\text{S}}=\left[\frac{\frac{S{_\text{MH}}\;\times\;S{_\text{C}}}{I{_\text{MH}}}-\frac{S{_\text{ML}}\;\times\;S{_\text{C}}}{I{_\text{ML}}}}{R{_\text{H}}-R{_\text{L}}}\times (R{_\text{S}}-R_\text{L)}+\frac{S{_\text{ML}}\times S{_\text{C}}}{I{_\text{ML}}}\right]\times \frac{I{_\text{MS}}}{W{_\text{S}}}\times D{_\text{S}}\times \frac{1000}{{M}_{\text{W}}}\times P$$

where *C*_S_ is the concentration of total testosterone in human serum (nmol/L).

**Table 1:** Uncertainty components of measurement of sample by aMCM

	Component	Subcomponent	Definition	Value	Unit	Standard uncertainty	Distribution pattern	PDF
1	*S*_MH_	/	Mass of standard working solution in standard blend 2	97.69	mg	0.01	Gaussian	N(97.69,0.01^2^)
2	*S*_ML_	/	Mass of standard working solution in standard blend 1	80.43	mg	0.01	Gaussian	N(80.43,0.01^2^)
3	The concentration of the standard working solution *S*_C_	*Q*_S1_	Mass of reference material in stock solution	3.63	mg	0.01	Gaussian	N(3.63,0.01^2^)
		*Q*_*ST1*_	Total mass of stock solution	7918.34	mg	0.005	Gaussian	N(7918.34,0.005^2^)
		*Q*_S2_	Mass of stock solution for dilute solution 1	804.21	mg	0.01	Gaussian	N(804.21,0.01^2^)
		*Q*_ST2_	Total mass of dilute solution 1	9084.56	mg	0.005	Gaussian	N(9084.56,0.005^2^)
		*Q*_S3_	Mass of dilute solution 1 for dilute solution 2	531.44	mg	0.01	Gaussian	N(531.44,0.01^2^)
		*Q*_ST3_	Total mass of dilute solution 2	46096.5	mg	0.005	Gaussian	N(46096.50,0.005^2^)
		*Q*_S4_	Mass of dilute solution 2 for working solution	928.28	mg	0.01	Gaussian	N(928.28,0.01^2^)
		*Q*_ST4_	Total mass of working solution	92286.98	mg	0.005	Gaussian	N(92286.98,0.005^2^)
4	*I*_MH_	/	Mass of internal standard solution in standard blend 2	88.52	mg	0.01	Gaussian	N(88.52,0.01^2^)
5	*I*_ML_	/	Mass of internal standard solution in standard blend 1	88.98	mg	0.01	Gaussian	N(89.98,0.01^2^)
6	*I*_MS_	/	Mass of internal standard solution in sample blend	88.20	mg	0.01	Gaussian	N(88.20,0.01^2^)
7	*W*_S_	/	Mass of sample	84.53	mg	0.01	Gaussian	N(84.53,0.01^2^)
8	*R*_H_	/	Peak isotope ratio of standard blend 2	1.1419	/	0.01759	Gaussian	N(1.1419,0.01759^2^)
9	*R*_L_	/	Peak isotope ratio of standard blend 1	0.94467	/	0.00911	Gaussian	N(0.94467,0.00911^2^)
10	*R*_S_	/	Peak isotope ratio of sample blend	1.00815	/	0.01185	Gaussian	N(1.00815,0.01185^2^)
11	*D*_S_	/	Density of sample	1.024	g/mL	0.0012	Rectangular	R(1.022,1.026)
12	*P*	/	Purity of reference material	94.90	%	0.95	Gaussian	N(0.949,0.0095^2^)
13	*M*_W_	/	Relative molecular weight of testosterone	288.4	g/moL	/	/	/

### Evaluating the probability density functions of the input quantities

According to the measurement model of testosterone in serum, Eq. (8), input quantities that have an influence on the measurand were obtained. The standard uncertainty, the distribution pattern, and the PDF of each input quantity were analyzed based on experimental data, literature, etc., shown in Table 1.

For the determination of total testosterone, the specific quality control serum sample was prepared and analyzed in two different runs, each run consisting of quintuplicate. *R*_L_, *R*_H_, and *R*_S_ were observed directly from instrument acquisition. The arithmetic means of each independent repeated observations of *R*_L_, *R*_H_, and *R*_S_ were used as the input estimate values to determine the measurement result. The experimental standard deviations of the means were used as the standard uncertainty of the input quantities by type A evaluation. The input quantities were described by the Gaussian distribution.

The standard working solution was prepared gravimetrically using certified reference material of testosterone by 4-step dilution. The uncertainty of the concentration of standard working solution *S*_C_ was related to eight individual uncertainty subcomponents according to the calibration data of the balance. The standard uncertainties of *S*_MH_, *S*_ML_, *I*_MH_, *I*_ML_, *I*_MS_, and *W*_S_ were also calculated according to the calibration data of the balance. The input quantities were described by a Gaussian distribution.

The density of the sample was 1.024 g/mL, and the expanded uncertainty was 0.002 g/mL. The input quantity was described by a symmetric rectangular distribution. According to the manufacturer’s specification, the purity of the certified reference material of testosterone (NMIA M914B) was 94.9% ± 1.9% (*k* = 2). The input quantity was described by a Gaussian distribution. The uncertainty of the relative molecular weight of testosterone *M*_W_ was taken no account.

### Propagation and summary information

The computer software MATLAB was used for the implementation of the propagation of probability distributions using aMCM. The MATLAB computer language was coded according to the PDFs shown in Table 1, listed in Supplementary Material.

**Fig. 1:**
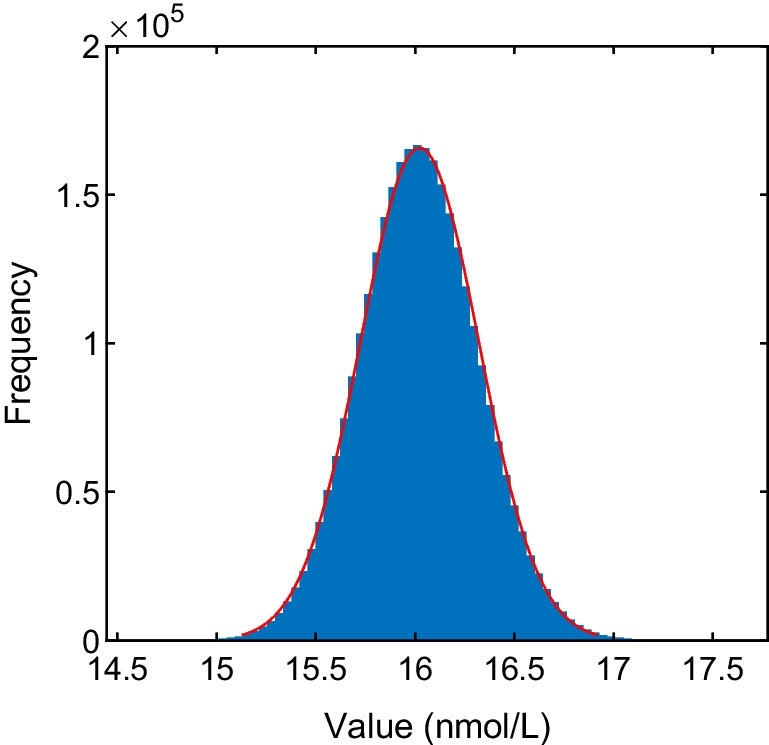
The histfit for output quantity *Y*

The *δ* was 0.005 as the number of significant decimal digits is 2 of the standard uncertainty of testosterone concentration. In order to ensure the reliability of the results, the *δ* was reduced to 0.001 [5]. The number of Monte Carlo trials was 2.974 × 10^6^ when the results have stabilized. The estimate of the testosterone concentration of 16.02 nmol/L, standard uncertainty of 0.2981 nmol/L, and the coverage interval of 15.45 to 16.62 nmol/L at coverage probability of 95% were obtained according to the aMCM procedure. The probability distribution for testosterone concentration was approximately described by a Gaussian distribution, shown as Fig. 1.

**Fig. 2:**
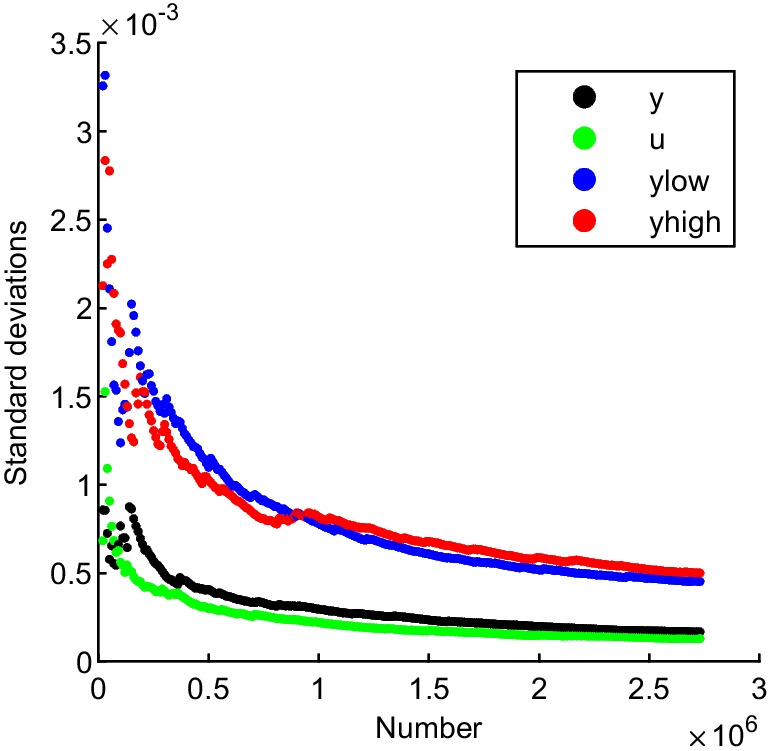
The relationship between the standard deviations and the number of *M*. y, the standard deviation associated with the average of the estimates *s*_*y*_; u, the standard deviation associated with the standard uncertainty *s*_*u*(*y*)_; ylow, the standard deviation associated with the left-hand endpoints of 95% coverage interval *s*_*y*low_; yhigh, the standard deviation associated with the right-hand endpoints of 95% coverage interval *s*_*y*high_

In order to analyze the relationship between the number of *M* and the standard deviations, a graph was drawn, shown as Fig. 2. The standard deviations were reduced rapidly to 0.002. The changes of the standard deviations were reduced with the increase of the number of *M*. The standard deviation of the associated standard uncertainty *u*(*y*) was stabilized fastest, and the standard deviation of the right endpoint of the coverage interval *y*_high_ was stabilized slowest.

### The result of validation of the GUM uncertainty framework

The uncertainty of testosterone concentration in the specific quality control serum sample was evaluated using GUM uncertainty framework according to our previous work [31]. The means, standard uncertainties, and 95% coverage intervals based on GUM uncertainty framework and aMCM were listed as Table 2. The results of total testosterone concentration obtained by two methods were consistent. The standard uncertainty obtained by GUM uncertainty framework was about 7% higher than the uncertainty by aMCM. The absolute differences *d*_low_ and *d*_high_ were larger than* δ*. The validation of results was not pass with the more narrower coverage interval obtained by aMCM. The probability distribution for testosterone concentration by aMCM was approximately described by a Gaussian distribution with a little right tail. The coverage interval obtained by aMCM was asymmetric. It may be the reason that the validation of results was not pass.

**Table 2:** The results associated with standard uncertainties by GUM and aMCM

Method	*δ*	*M*	*y*	*u*_*y*_	95%CI	|*d*_low_|	|*d*_high_|	Validation of results (*δ* = 0.001)
GUM	/	/	16.019	0.320	15.392 ~ 16.646	0.056	0.026	Not pass
aMCM	0.001	2.974 × 10^6^	16.023	0.298	15.448 ~ 16.620			
	0.001	3.03 × 10^6^	16.023	0.299	15.448 ~ 16.620			
	0.005	1.4 × 10^5^	16.023	0.298	15.447 ~ 16.620			

Two applications of the aMCM with *δ* of 0.001, different random samplings from the PDFs, were made to demonstrate the variation in the results obtained. The results were consistent within the numerical tolerance used and the numbers of *M* were slightly different. Consistent with the guidance [5], the required number of *M* with a numerical tolerance of *δ*/5 was approximately 25 times that with a numerical tolerance of *δ*.

## Discussion

The identification of the major uncertainty components is very important for the assessment and the optimization of the measurement procedure to reduce the uncertainty. According to the analysis of the relevant input quantities listed in Table 1, the purity of reference material *P*, the peak isotope ratios (*R*_L_, *R*_H_, and *R*_S_), and the mass of reference material in stock solution have the great influences on the uncertainty of testosterone concentration.

With prescribed *δ* and coverage probability, the number of *M* has a big impact on the quality of the numerical results. A number of *M* = 10^6^ often be expected to deliver a coverage interval at coverage probability of 95% for the output quantity. The quality control of the numerical results is not provided by MCM. A procedure that selects *M* adaptively is more appropriate as the various results of interest have stabilized in a statistical sense.

GUM uncertainty framework need the measurement model to be linear or linearizable and the characterization of the measurand to be a Gaussian distribution or a scaled and shifted *t*-distribution that limit its application. As a practical alternative to the GUM uncertainty framework, the MCM is an extremely appropriate tool to perform uncertainty calculations and has wider application than the GUM uncertainty framework. The numerical results by MCM are valid, especially for a complex model function as well as for arbitrary PDFs assigned to the input quantities, since it does not make approximating assumptions. MCM may be easier to apply than GUM uncertainty framework due to difficulties in calculating sensitivity coefficients. MCM also be applied to assess the quality of those provided by the GUM uncertainty framework.

The probability distribution for testosterone concentration by aMCM was approximately described by a Gaussian distribution with a little right tail, and the coverage interval obtained by MCM was asymmetric. It may be the reason that the validation of GUM was not pass and the right endpoint of the coverage interval *y*_high_ was stabilized slowest. The results provided by the GUM uncertainty framework might be unreliable in this case. The standard uncertainty result obtained by the aMCM slightly lower about 7% than that obtained by GUM uncertainty framework in this work. This difference is may not important when considering decision related to the “fitness for purpose” of the conventional measuring procedure. The measurement uncertainty of the high-order RMP should be evaluated more carefully. The MCM is recommended to use for the uncertainty evaluation of the high-order RMPs or as a validation method of the GUM uncertainty framework.

In conclusion, the aMCM was first proposed to estimate the measurement uncertainty of the RMP for total testosterone in human serum based on triple isotope dilution liquid chromatography tandem mass spectrometry and was used for the validation of the GUM uncertainty framework.

## Supplementary Information

Below is the link to the electronic supplementary material.

Supplementary file1 (PDF 169 KB)
