# Supplementary material for: Application of the adaptive Monte Carlo method for uncertainty evaluation in the determination of total testosterone in human serum by triple isotope dilution mass spectrometry
Source: Anal Bioanal Chem. 2024 Jun 19;416(19):4427–34. doi: 10.1007/s00216-024-05380-z (PMC11525383; doi:10.1007/s00216-024-05380-z)
Supplement: Supplementary file 1 — Supplementary file1 (PDF 169 KB) [file 216_2024_5380_MOESM1_ESM.pdf]

Supplementary Material

Application of the adaptive Monte Carlo method for uncertainty evaluation in the determination of total testosterone in human serum by triple isotope dilution mass spectrometry

Gongcheng Liu[a, 1], Huimin Wang[b, 1], Yanlin Han[a], Chunlong Liu[a] and Man Liang[a]*

[1]*These authors contributed equally to this research*

[a] *Reference Laboratory, Autobio Diagnostics Co., Ltd, Zhengzhou, Henan 450016, China*

[b] *Department of Laboratory Medicine, Affiliated Hospital of Nantong University, Nantong, Jiangsu 226001, China*

* Corresponding author: Man Liang. E-Mail: liangman@autobio.com.cn

A reference measurement procedure for the analysis of testosterone in human serum, based on isotope dilution liquid chromatography tandem mass spectrometry with electrospray ionization, has been performed and critically evaluated. The nomination of the RMP for reference measurement services had been reviewed by the JCTLM and was recently approved for listing in the JCTLM Database. The report for the details of isotope dilution mass spectrometry procedure and the data of method validation was provided in the JCTLM Database. The adaptive Monte Carlo procedure and the MATLAB computer language are included in the Supplementary Material.

Estimation of expanded uncertainty

The adaptive Monte Carlo procedure

The adaptive Monte Carlo procedure is carried out a sequence as sollows:

a) Set n_{dig}, denoting the number of significant decimal digits regarded as meaningful in a numerical value z, to an appropriate small positive integer. Normally n_{dig} would be chosen to be 1 or 2. z is expressed in the form $c \times 10^{\ell}$, where c is an n_{dig} decimal digit integer and ℓ an integer. The numerical tolerance δ is calculated according to the following equation.

$$\delta = \frac{1}{2} 10^{\ell}$$

b) Set M = 10000.

c) Set h = 1, denoting the first application of MCM in the sequence.

d) Carry out M Monte Carlo trials. With $x^{(j)}_1, x^{(j)}_2, \ldots, x^{(j)}_N, j=1,\ldots,M$, the model values Y are obtained from the following equation. The model values, $y^{(j)}$, provided by MCM are sorted into non-decreasing order, if necessary, small numerical perturbations are performed on all repeated model output values.

$$y^{(j)} = f(x_1^{(j)}, x_2^{(j)}, \ldots, x_N^{(j)})$$

e) Calculate $y(h)$, $u(y(h))$ as an estimate of Y, the associated standard uncertainty according to the following equations. The left- and right-hand endpoints of a 95% coverage interval, $y(h)_{\mathrm{low}}$ and $y(h)_{\mathrm{high}}$, are obtained by taking the 2.5 and 97.5 percentiles of the sample order.

$$\bar{y} = \frac{1}{M} \sum_{j=1}^{M} y^{(j)}$$

$$u(y) = \sqrt{\frac{1}{M-1} \sum_{j=1}^{M} (y^{(j)} - \bar{y})^2}$$

f) If h = 1, increase h by one and return to step c).

g) Calculate the standard deviation s_y associated with the average of the estimates $y(1), \ldots, y(h)$ of Y, given by the following equation.

$$s^2{}_y = \frac{1}{h(h-1)} \sum_{r=1}^{h} (y(r) - y)^2$$

where

$$y = \frac{1}{h} \sum_{r=1}^{h} y(r)$$

h) Calculate the counterpart of this statistic for $u(y)$, y_{low} and y_{high}.

i) use all $h \times M$ model values available so far to form $u(y)$.

j) if any of $2s_y$, $2s_{u(y)}$, $2s_{y\mathrm{low}}$ and $2s_{y\mathrm{high}}$ exceeds δ, increase h by one and return to step c).

k) Regard the overall computation as having stabilized, and use all $h \times M$ model values obtained to calculate y, $u(y)$ and a coverage interval.

The matlab computer language

The matlab computer language is coded as follows:

```
h=1;
e=0.001;
y=1;
u=1;
ylow=1;
yhigh=1;
Ma=0;
Sa=0;
YL=0;
YH=0;
w=0;
while(2*y>e|2*u>e|2*ylow>e|2*yhigh>e)
M=10000;
QS1= random('norm',3.63,0.01,1,M);
QST1= random('norm',7918.34,0.005,1,M);
QS2= random('norm',804.21,0.01,1,M);
QST2= random('norm',9084.56,0.005,1,M);
QS3= random('norm',531.44,0.01,1,M);
QST3= random('norm',46096.50,0.005,1,M);
QS4= random('norm',928.28,0.01,1,M);
QST4= random('norm',92286.98,0.005,1,M);
SMH= random('norm',97.69,0.01,1,M);
IMH= random('norm',88.515,0.01,1,M);
SML= random('norm',80.43,0.01,1,M);
```

```
IML= random('norm',88.98,0.01,1,M);
RH= random('norm',1.1419,0.0175907172489735,1,M);
RL= random('norm',0.944666666666667,0.0091087703324706,1,M);
RS= random('norm',1.00815,0.0118467183997133,1,M);
IMS= random('norm',88.195,0.01,1,M);
WS= random('norm',84.53,0.01,1,M);
DS= random('unif',1.022,1.026,1,M);
P= random('norm',0.949,0.0095,1,M);
SC=QS1./QST1.*QS2./QST2.*QS3./QST3.*QS4./QST4.*1000000000;
ML=SML.*SC./IML;
MH=SMH.*SC./IMH;
Y= ((MH-ML).*(RS-RL)./(RH-RL)+ ML).*IMS.*DS.*P./WS./288.4*1000;
if h~=1
    w=horzcat(w,Y);
else
    w=Y;
end
Ma(i)=mean(Y);
Sa(i)=std(Y);
YL(i)=prctile(Y,2.5);
YH(i)=prctile(Y,97.5);
if h~=1
    y=std(Ma)/sqrt(i);
    u= std(Sa)/sqrt(i);
    ylow=std(YL)/sqrt(i);
    yhigh= std(YH)/sqrt(i);
end
h=h+1;
end
  [mean(w),std(w),prctile(w,2.5),prctile(w,97.5)]
```

[i]

histfit(w,80)
